# Sleep deprivation in third-trimester pregnancies is multi-causal and requires careful differential consideration

**DOI:** 10.1590/1806-9282.20241690

**Published:** 2025-06-16

**Authors:** Carla Alexandra Scorza, Fulvio Alexandre Scorza, Josef Finsterer

**Affiliations:** 1 Universidade Federal de São Paulo, Escola Paulista de Medicina – São Paulo (SP), Brazil.; 2 Neurology and Neurophysiology Center, Department of Neurology – Vienna, Austria.

Dear Editor,

We were interested to read the article by Kaya et al. on a cross-sectional study of the factors influencing sleep quality in 570 women in the third trimester of pregnancy using the Pittsburgh Sleep Quality Index (PSQI), the International Physical Activity Questionnaire-Short Form (IPAQ-SF), the Restless Leg Syndrome Form (RLSF), the Brief Fatigue Inventory (BFI), and the Perceived Stress Scale (PSS)^1^. The mean PSQI was 6.0, and half of the patients had a PSQI >5^1^. Sleep quality in the cohort was determined by hemoglobin levels; smoking status; leg, back, hip, or neck pain; restless leg syndrome (RLS); level of fatigue; stress; income; and attendance at antenatal schools^1^. It was concluded that sleep quality can be improved in the third trimester of pregnancy by minimizing the identified risk ­factors for sleep disturbances^1^. The study is ­convincing, but some points should be discussed.

The first point is that the range of factors that determine sleep quality in third-trimester pregnancies is much broader than assumed. Sleep quality in these patients may also be highly dependent on various other internal and external factors, such as diet quality and composition (coffee, tea, Red Bull, and alcohol); the timing of last meal and last drink before sleep; frequency of nocturia; personality type, comorbidities ­including psychiatric disorders (e.g., attention-deficit/­hyperactivity ­disorder, obsessive-compulsive disorder, mood disorders, anxiety disorders, and psychosis) and hormonal disorders (e.g., hyperthyroidism and diabetes); heart or lung disease pain; sweating; concomitant medications; condition of the sleeping environment [brightness (e.g., TV, cell phone, laptop, and tablet), noise (e.g., street, neighbors, refrigerator, air conditioning, ventilation, animals, alarm clock, radio, TV, and cell phone), electrosmog, ­humidity, temperature, and ventilation]; air ­pollution; and body mass index. These factors need to be included in the analysis before final conclusions can be drawn about the factors that influence the sleep quality of pregnant women in the third trimester.

The second point is that we disagree with the statement that iron deficiency is the strongest risk factor for RLS^1^. More often than iron deficiency, RLS is due to polyneuropathy. One of the most common causes of polyneuropathy is diabetes^2^. Since diabetes is common in pregnant women, it should be considered a cause of polyneuropathy and thus RLS. How many of the included patients had a history of diabetes or developed gestational diabetes? How many of the included patients had both diabetes and RLS?

The third point is that the number of pregnancies was not included in the assessment^1^. Multipara have better sleep quality than primipara^3^, for whom the situation is new and uncertain, while multipara are familiar with the situation and may have developed effective coping strategies to prevent the stress from becoming too great.

The fourth point is that the impact of sleep disturbance on pregnancy outcomes has not been studied. Were women with severe sleep disturbances more likely to have stillbirths, preterm births, premature birth, or emergency cesarean sections?

Overall, this interesting study has limitations that put the results and their interpretation into perspective. Addressing these limitations could strengthen the conclusions and ­support the study's message. The risk factors for sleep deprivation in third-trimester women are multiple and should be included in the assessment of risk factors and management of sleep ­disorders in this cohort.
